# Comparison of efficacy and safety of complementary and alternative therapies for coronary heart disease complicated with anxiety or depression disorder: A protocol for Bayesian network meta-analysis

**DOI:** 10.1097/MD.0000000000025084

**Published:** 2021-03-26

**Authors:** Xueyan Han, Xinxin Liu, Fengxing Zhong, Yiguo Wang, Hui Guan, Qiming Zhang

**Affiliations:** a First College of Clinical Medicine, Shandong University of Traditional Chinese Medicine, Jinan, Shandong Province; b Experimental Research Center, China Academy of Chinese Medical Sciences, Beijing; c Shandong University of Traditional Chinese Medicine, Jinan, Shandong Province, China.

**Keywords:** anxiety, Bayesian, complementary and alternative therapies, coronary heart disease, depression, network meta-analysis, protocol

## Abstract

**Background:**

With the acceleration of the pace of life, the phenomenon of anxiety and depression in patients with coronary heart disease (CHD) is more and more common, and “psycho-cardiology” arises spontaneously. At present, the drug treatments of psycho-cardiology are difficult to achieve satisfactory results, and the side effects are obvious. Complementary and replacement therapies of CHD complicated with anxiety or depression disorder play an increasingly positive role, but there is a lack of comparison among different complementary and alternative therapies. In this study, Bayesian network meta-analysis (NMA) analysis method will be used for the first time to synthesize all the evidences of direct and indirect comparison among a variety of interventions, and rank their effectiveness and safety.

**Methods:**

Two independent researchers will search from the beginning to January 2021 mainly including randomized controlled trials (RCTs) and closely related ongoing RCTs of complementary and alternative therapies for CHD complicated with anxiety or depression disorder. And then identify, select and extract the data. The primary outcome measures are frequency of acute attack angina, severity of angina pectoris; the changed score in the validated scales, which can assess severity of anxiety or depression. Secondary outcomes include total efficacy rate, electrocardiogram improvement, traditional Chinese medicine symptoms score, changes of dosage of nitroglycerin and adverse effects. Using softwares WinBUGS 1.4.3 and STATA 16.0 for pairwise meta-analysis and NMA to comprehensively evaluate various interventions. The quality of evidences will be evaluated through the Grading of Recommendations Assessment, Development and Evaluation.

**Results:**

This NMA will comprehensively compare and rank the efficacy and safety of a series of complementary and alternative therapies in the treatment of CHD complicated with anxiety or depression disorder.

**Conclusion:**

Supplementary and replacement therapies play an essential role in improving CHD complicated with anxiety or depression disorder. We expect that the NMA will provide reliable evidences of evidence-based medicine for treatment of CHD complicated with anxiety or depression disorder.

**Protocol registration number:**

INPLASY202120046.

**Ethical approval:**

This review does not require ethical approval.

## 1 Introduction

“Psycho-cardiology” is a new concept for the treatment of coronary heart disease (CHD) that has been popularized in clinic in recent years. The development of psycho-cardiology can be traced back to the early 19th century, when German psychiatrist Heinroth put forward the concept of psychosomatic disease. In the 1930 s, Dr Dumbar put forward the scientific concept of psychosomatic medicine and psychosomatic diseases, also known as “psychophysiological disorders” on the basis of experiments.^[1]^ In 1985, James W.Jefferson,^[2]^ mental medical college, Wisconsin University of the United States, published an article in Psychosomatic, and for the first time put forward a new term “psychocardiology,” to lay the foundation for the study of “psycho-cardiology” in countries all over the world. Psycho-cardiology mainly studies the relationship between psychological diseases and cardiovascular diseases.

CHD is a common and frequently-occurring disease in cardiovascular department, with a high fatality rate.^[3–5]^ In the past, the prevention and treatment of CHD tended to interfere with its traditional biological risk factors, such as atherosclerosis, hypercholesterolemia, insulin resistance, hyperhomocysteinemia and so on. The application of vascular recanalization methods such as nitrates, anti-platelet aggregation, anticoagulation and antifibrinolysis and percutaneous coronary intervention has also made a breakthrough in the treatment of acute coronary syndrome. In spite of this, there are still some patients with CHD who do not respond well to the above treatment measures, and medical workers gradually realize that there may be other risk factors for CHD besides biological factors. A number of evidence-based medicine studies have showed that psychosocial factors are involved in the occurrence and development of CHD.^[6,7]^ With the increasingly fierce social competition and the increasing pressure of work and life, anxiety or depression is more and more common in patients with CHD. The EmdinCA team^[8]^ conducted a meta-analysis of 46 cohort studies, which were combined into 2017276 normal subjects and 222253 anxiety patients. The final results showed that anxiety was highly correlated with cardiovascular mortality (RR 1.41, confidence interval [CI] 1.13–1.76) and significantly related to the incidence of CHD (RR 1.41, CI 1.23–1.61). Based on the study of the incidence of psychological disorders in patients after percutaneous coronary intervention, foreign scholars found that patients with mild anxiety, moderate anxiety and severe anxiety accounted for 41.32%, 28.54%, and 6.41%, respectively. During the postoperative follow-up, only 10.84% of the patients with moderate anxiety were relieved.^[9]^ A meta-analysis in 2004 found that patients with depression were more likely to develop CHD after excluding other interfering items, about 1.5 to 2.0 times higher than those without depression, and patients with CHD coexisting depression had a significantly increased risk of long-term cardiovascular events, about 2.0 to 2.5 times higher than those without coexisting depression.^[10]^ The American Heart Association reported that about 20% of hospitalized patients with acute coronary syndrome meet the diagnostic criteria for depression, and more patients may have varying degrees of depressive symptoms.^[11]^ Clinical studies suggested that the incidence of CHD with depression was 17% to 27%.^[12]^ A clinical study of 125 patients with CHD (age 64.2 ± 4.7 years old) showed that 80 patients (64.3%) were associated with different degrees of depression, and the severity of depression was related to age, sex, complications and other factors.^[13]^ The western medicine treatment of “psycho-cardiology” abnormality of CHD is mainly the combination of anti-angina pectoris drugs and anti-anxiety drugs or antidepressants. However, anti-anxiety drugs or antidepressants have certain cardiotoxicity, and their combination will often cause adverse reactions such as bradycardia and myocardial ischemia, and may even worsen the long-term prognosis of CHD.^[14,15]^ As a result, more and more patients with CHD coexisting anxiety or depression disorder are looking for ways other than drugs to improve their symptoms.^[16–18]^

In recent years, complementary and replacement therapies, such as traditional Chinese medicine (TCM), are more and more widely used in psycho-cardiology, with remarkable efficacy and few side effects, so it has a broad prospect. Its therapeutic concept of “integration of form and spirit” has a unique advantage in the diagnosis and treatment of CHD with “psycho-cardiology” abnormality. In addition, cognitive-behavioral therapy, exercise therapies (yoga, Tai Chi, Five-Animal Frolics Exercise and so on), music therapy and relaxation training are also complementary and alternative therapies for psycho-cardiology and the efficacy is definite. In a double-blinded randomized controlled trials (RCTs), involving 60 patients with anxiety or depression symptoms, Huan Ma et al demonstrated that TCM Xinkeshu tablets could affect the blood ratio of anti-inflammatory:pro-inflammatory cytokines and effectively control the symptoms related to anxiety and depression of patients with CHD.^[19]^ Jun Jiang et al found that Five-Animal Frolics Exercise intervention could improve the anxiety and depression symptoms in CHD patients and their life quality by adjusting serum levels of miR-124 and miR-135.^[20]^ A systematic review indicated that listening to music had a beneficial effect on anxiety in persons with CHD.^[21]^

Although there have been many RCTs and systematic reviews to evaluate the efficacy of interventions, it is still difficult to choose from a large number of comparative evidences. Traditional meta-analysis is based on a direct comparison of strictly designed RCTs and has been recognized as the most convincing evidence to evaluate the efficacy of interventions. However, for the indirect comparison of many intervention measures to treat the same disease and to select the safest and most effective measures for patients, the traditional meta-analysis seems powerless at this time. The network meta-analysis (NMA) can just solve this problem.

## 2 Methods

The protocol will be completed following the Preferred Reporting Items for Systematic Review and Meta-Analysis Protocols guidelines.^[22]^

### 2.1 Study registration

The protocol was registered on the International Platform of Registered Systematic Review and Meta-analysis Protocols (INPLASY). No: INPLASY202120046. (URL = https://inplasy.com/inplasy-2021–2-0046/)

### 2.2 Inclusion criteria

#### 2.2.1 Type of study

All RCTs and systematic reviews/meta-analysis relating to complementary and alternative therapies for patients suffering CHD complicated with anxiety or depression disorder will be screened out.

#### 2.2.2 Participants

(1) Patients diagnosed the CHD, including angina pectoris of stable and unstable, coexisting anxiety or depression disorder;
(2) There are no age, gender, region or race restrictions.

#### 2.2.3 Interventions

The control group used basic treatment of anti-angina pectoris with or without western medicine of anti-anxiety drugs or antidepressants. The experimental group was treated with complementary and alternative therapies including Chinese herbal medicine, acupuncture, massage, relaxation training, music therapy, cognitive-behavioral therapy, exercise therapies (yoga, Tai Chi, Five-Animal Frolics Exercise and so on) on the basis of treatment in the control group. All kinds of complementary and alternative therapies can be used alone or in any combination.

#### 2.2.4 Outcome measures

The frequency of acute attack angina, severity of angina pectoris; the change in the Hamilton Anxiety Rating scale score (with higher scores indicating more depression), Hamilton Depression Rating scale score, or Zung Self-Rating Anxiety Scale score, Zung Self-Rating Depression Scale score, or any other validated scales, which can assess severity of anxiety or depression are primary outcome measures. Secondary outcomes include total efficacy rate, electrocardiogram improvement, TCM symptoms score, changes of dosage of nitroglycerin and adverse effects.

### 2.3 Exclusion criteria

(1) The publications containing cardiac neurosis, heart failure or severe arrhythmia in the inclusion criteria will be excluded;
(2) Those with unclear outcome measures, no assessing response of angina pectoris or anxiety and depression will be excluded;
(3) Exclusion of incomplete RCTs, including semi-RCTs, case reports, reviews, animal experiments and the studies that lack of efficacy contrast before and after in the control group;
(4) Repeatedly published literature, or literature with similar data.

### 2.4 Databases and search strategy

Our search from the beginning to January 2021 mainly by two researchers include English databases such as PubMed, EMBASE, Cochrane Library, Cochrane Central Register of Controlled Trials, Web of Science, et al and Chinese databases such as VIP full-text database, CNKI, Wanfang Database, including all RCTs of complementary and alternative therapies of CHD complicated with anxiety or depression disorder. Besides that, closely related ongoing RCTs in the ClinicalTrials.gov and World Health Organization International Clinical Trials Registration Platform (WHO ICTRP) will be searched. We will also track the references of searched systematic review or meta-analysis. If there is a disagreement in the process of literature screening, it can be discussed by two researchers and then decide whether to include it or not. If no agreement can be reached, it can be judged by the intervention of a third researcher. We will use the medical subject headings combined with free words to retrieve literature. Table 1 shows the preliminary search strategy for PubMed, and we will adjust it appropriately according to the specific situation.

**Table 1:** Draft of search strategy for PubMed.

NO.	Search item
#1	Coronary Heart Disease[MeSH]
#2	Coronary Heart Disease[Title/Abstract] OR Coronary Artery Disease[Title/Abstract] OR Angina Pectoris[Title/Abstract] OR Myocardial Infarction[Title/Abstract] OR Acute Coronary Syndrome[Title/Abstract] OR Cardi∗ [Title/Abstract] OR ACS[Title/Abstract] OR psycho-cardiology[Title/Abstract] OR psychocardiology[Title/Abstract].
#3	#1 OR #2
#4	Anxiety[MeSH]
#5	Anxiety[Title/Abstract] OR Anxiety Disorder[Title/Abstract] OR Anxious Symptoms[Title/Abstract] OR Anxious Symptom[Title/Abstract] OR Symptom, Anxious[Title/Abstract] OR Symptoms, Anxious[Title/Abstract] OR Emotional Anxiety[Title/Abstract] OR Anxiety, Emotional[Title/Abstract] OR Anxieties, Emotional[Title/Abstract] OR Emotional Anxieties[Title/Abstract]
#6	#4 OR #5
#7	Depression[MeSH]
#8	Depressions[Title/Abstract] OR Depression Disorder[Title/Abstract] OR Depressive Symptoms[Title/Abstract] OR Depressive Symptom[Title/Abstract] OR Symptom, Depressive[Title/Abstract] OR Symptoms, Depressive[Title/Abstract] OR Emotional Depression[Title/Abstract] OR Depression, Emotional[Title/Abstract] OR Depressions, Emotional[Title/Abstract] OR Emotional Depressions[Title/Abstract]
#9	#7 OR #8
#10	#3 AND #6
#11	#3 AND #9
#12	#10 OR #11
#13	Complementary Therapies[MeSH]
#14	Therapy, Alternative[Title/Abstract] OR Therapy, Complementary[Title/Abstract] OR Complementary Therapies[Title/Abstract] OR Alternative Therapies[Title/Abstract] OR Medicine, Alternative [Title/Abstract] OR Alternative Medicine[Title/Abstract] OR Complementary Medicine [Title/Abstract] OR Medicine, Complementary[Title/Abstract] OR Herbal Therapy[Title/Abstract] OR Complementary and alternative therapies[Title/Abstract]
#15	#13 OR #14
#16	Chinese herbal medicines[Title/Abstract] OR Traditional Chinese Medicine[Title/Abstract] OR Acupuncture [Title/Abstract] OR relaxation training [Title/Abstract] OR Massage [Title/Abstract] OR cognitive-behavioral therapy [Title/Abstract] OR Exercise [Title/Abstract] OR Massage [Title/Abstract] OR Five-Animal Frolics Exercise[Title/Abstract] OR Music therapy [Title/Abstract] OR Yoga [Title/Abstract] OR Tai chi [Title/Abstract]
#17	#15 OR #16
#18	Randomized Controlled Trial [Publication Type] OR Controlled Clinical Trial [Publication Type] OR Randomized [Title/Abstract] OR randomly [Title/Abstract]
#19	#12 AND #17 AND #18

ACS = acute coronary syndrome, MeSH = medical subject headings.

### 2.5 Data extraction

Preliminary screening: through the same search pattern, two researchers independently exclude the literature obtained by reading literature titles and abstracts that does not meet the inclusion criteria, and collect the literature obtained after the initial screening. The coincident literature will be retained a copy to be included, and the disagreement literature will be discussed by the two researchers to decide whether to include it or not. If no agreement can be reached, the third party will intervene to judge.

Full-text screening: download the full text of the qualified studies after preliminary screening, and the two researchers independently read and evaluate the full text of the same studies, and further establish whether the studies meet the inclusion criteria of this NMA. At the same time, the causes for literature exclusion are recorded. Like the preliminary screening, after the two researchers complete the literature independently, those with overlapping views will be included, and those with different opinions will be discussed, and then if the results still are inconsistent, the third party can evaluate whether they are included or not. The following information of the literature will be extracted:

#### 2.5.1 General information

Title, first author, country, year, journal, the support of the fund, the sources of the original literature;

#### 2.5.2 Methodological materials

Randomization procedure, inclusion criteria, exclusion criteria, sample size, people lost to follow-up or dropped out, security, ethics;

#### 2.5.3 Research objects

Age, race, gender, sources of participants, course of disease;

#### 2.5.4 Intervention measures and observation indicators

The methods of intervention, dosage, course of treatment, primary and secondary outcome measures, and follow-up time.

If the information of the article is incomplete, the required information will be got through contacting the original author.

### 2.6 Risk of bias assessment

In this study, the Cochrane Collaboration's Risk of Bias Tool will be used to evaluate the bias risk of the included literature, which evaluates the bias risk of the literature from aspects of random method, assignment concealment, blind method, outcome data integrity, selective report, number of dropped cases, follow-up, and other biases.^[23]^ The three levels - low bias risk, unclear bias risk and high bias risk, will be used to evaluate each of the above aspect and finally the bias risk will be summarized.

### 2.7 Assessment of heterogeneity

Test the heterogeneity of multiple research results and merge the data with good homogeneity. The appropriate effect model should be selected according to the results of heterogeneity analysis.

In this study, *I*^*2*^ value and *P*-value are used to test the statistical heterogeneity among studies. When *I*^*2*^ ≤ 50% and *P* > .1, the fixed effect model can be used. When *I*^*2*^ > 50% and *P* ≤ .1, then according to the causes of heterogeneity, explore the source of heterogeneity, and then carry out subgroup analysis and merge statistics. If there is still heterogeneity among studies after analysis, then the random effect model can be used.

### 2.8 Subgroup analysis

When there is heterogeneity among studies, the sources of heterogeneity will be explored and all the data will be divided into smaller units so that they can be compared in each subgroup.

### 2.9 Sensitivity analysis

Sensitivity analysis is an analytical method that determines the sensitivity of a study result or how some important factors change NMA. For example: using different statistical methods to re-analyze the data, such as using random effect model instead of fixed effect model; re-analyzing the data after reasonable estimation of the missing data; from the inclusion studies to exclude the literature with relatively poor quality and re-analysis, before and after comparison, whether there is a significant difference.

## 3 Statistical analysis

### 3.1 Pairwise meta-analysis

STATA16.0 software is used for pairwise meta-analysis. The qualitative data are grouped into bivariate variables and then merged, and the quantitative data are merged by mean difference. The results are expressed by odds ratio and mean difference respectively. 95% CI is employed for interval estimation. *I*^2^ value as a measure is used to test the degree of heterogeneity among studies.

### 3.2 Network meta-analysis

Stata 16.0 will be used to draw the network plots, forest plots, rank probability diagrams, comparison-correction funnel chart of publication bias and corresponding statistics.^[24]^ WinBUGS 14 software will be used for statistical analysis and based on Bayesian theory, the Markov chain Monte Carlo (MCMC) random effect model will be used for NMA.^[25]^ The number of iterations will be set to 50000 times, and the first 10000 times are used for annealing to eliminate the influence of the initial value, and then the last 40000 times are used for sampling. The Brooks-Gelman-Rubin statistical method will be used to evaluate the convergence of the model. The intervention measures will be ranked by the surface under the cumulative ranking curve values.^[26]^ The higher the value of surface under the cumulative ranking curve values, the better the effect of the intervention measures.

### 3.3 Assessment of inconsistency

The node splitting method will be used for consistency test. If the difference is not statistically significant (*P* > .05), it suggests that the results of direct comparison and indirect comparison is consistent,^[27]^ and Stata16.0 will be used to assess the consistency of each closed loop. The consistency of each closed loop is evaluated by inconsistency factors and its 95% CI, and the 95% CI containing 0 is considered to be consistent, otherwise it is considered that there is obvious inconsistency in the closed loops.

## 4 Evaluation of publication bias and evidence quality

If more than 10 articles are included, the significant publication bias will be identified by drawing the comparison-correction funnel chart. Most of the dots are distributed on one side of the central axis, suggesting that there may be a serious publication bias. If there is no obvious publication bias, the dots in the funnel plot are symmetrically and uniformly distributed on both sides of the central axis.

The level of evidences and recommendation intensity are evaluated through Grading of Recommendations Assessment, Development and Evaluation. In NMA, the quality of evidences includes the 5 grades of the risk of bias, indirectness, inconsistency, imprecision, and publication bias.^[28]^

## 5 Discussion

“Psycho-cardiology” abnormality of CHD refers to a kind of comprehensive disease of CHD complicated with mental and psychological diseases. Modern studies have found that, on the one hand, psychological stress can lead to myocardial ischemia and worsen the prognosis of patients with CHD and then the risk of cardiovascular events is significantly higher. On the other hand, patients with CHD are easy to show bad psychological states such as anxiety and depression because of the cognitive impairment of the disease. The 2 cause and effect each other, forming a vicious circle, which is not conducive to the prognosis of the disease.^[29–31]^

At present, the efficacy of drug treatment of CHD complicated with anxiety or depression disorder cannot meet the needs of people and the side effects are obvious. In the traditional systematic review of complementary and alternative therapies, it has been proved that complementary and replacement therapies of CHD complicated with anxiety or depression disorder play an increasingly positive role, but there is a lack of direct comparison among different complementary and alternative therapies. It is not beneficial to clinical application and the choice of the best treatment. This study will systematically search the literature, and using Bayesian NMA analysis method for the first time, synthesize all the evidences of direct and indirect comparison among a variety of intervention measures, and then rank their effectiveness and safety, in order to provide reference for clinical treatment of CHD complicated with anxiety or depression disorder.

However, this study also has some limitations. For example, due to the limitations of the original study, we are unable to evaluate the long-term efficacy of complementary and alternative therapies in the treatment of CHD complicated with anxiety or depression. Nevertheless, we still expect that the NMA will provide reliable evidences of evidence-based medicine for treatment of CHD complicated with anxiety or depression disorder, and to a certain extent, provide some new insights into the complementary and alternative therapies of CHD complicated with anxiety and depression disorder.

## Author contributions

**Conceptualization:** Xueyan Han, Xinxin Liu, Qiming Zhang

**Formal analysis:** Xueyan Han, Xinxin Liu

**Methodology:** Fengxing Zhong, Yiguo Wang

**Project administration**: Qiming Zhang

**Software:** Xueyan Han, Hui Guan

**Writing – original draft**: Xueyan Han, Xinxin Liu

**Writing – review & editing**: Qiming Zhang, Xueyan Han
